# Use of oxytocin during Caesarean section at Princess Marina Hospital, Botswana: An audit of clinical practice

**DOI:** 10.4102/phcfm.v5i1.418

**Published:** 2013-02-26

**Authors:** Billy M. Tsima, Farai D. Madzimbamuto, Bob Mash

**Affiliations:** 1 Division of Family Medicine and Primary Care, Stellenbosch University, South Africa; 2 Department of Anaesthesia and Critical Care, University of Botswana, Botswana; 3 Department of Family Medicine, University of Botswana

## Abstract

**Background:**

Oxytocin is widely used for the prevention of postpartum haemorrhage. In the setting of Caesarean section (CS), the dosage and mode of administrating oxytocin differs according to different guidelines. Inappropriate oxytocin doses have been identified as contributory to some cases of maternal deaths. The main aim of this study was to audit the current standard of clinical practice with regard to the use of oxytocin during CS at a referral hospital in Botswana.

**Methods:**

A clinical audit of pregnant women having CS and given oxytocin at the time of the operation was conducted over a period of three months. Data included indications for CS, oxytocin dose regimen, prescribing clinician's designation, type of anaesthesia for the CS and estimated blood loss.

**Results:**

A total of 139 case records were included. The commonest dose was 20 IU infusion (31.7%). The potentially dangerous regimen of 10 IU intravenous bolus of oxytocin was used in 12.9% of CS. Further doses were utilized in 57 patients (41%). The top three indications for CS were fetal distress (36 patients, 24.5%), dystocia (32 patients, 21.8%) and a previous CS (25 patients, 17.0%). Estimated blood loss ranged from 50 mL – 2000 mL.

**Conclusion:**

The use of oxytocin during CS in the local setting does not follow recommended practice. This has potentially harmful consequences. Education and guidance through evidence based national guidelines could help alleviate the problem.

## Abstrait

**L'utilisation d'ocytocine au cours des césariennes à l'hôpital Princess Marina, au Botswana: audit de pratique clinique**

**Contexte:**

L'ocytocine est couramment utilisée pour la prévention de l'hémorragie post-partum. Lors d'une césarienne, le dosage et le mode d'administration de l'ocytocine diffèrent d'une directive à l'autre. Des doses inappropriées d'ocytocine ont été identifiées comme contribuant à certains cas de décès maternels. Le principal objectif de cette étude était d’évaluer la norme actuelle de pratique clinique concernant l'utilisation d'ocytocine pendant une césarienne dans un hôpital de référence au Botswana.

**Méthodologie:**

Un audit clinique de femmes enceintes ayant subi une césarienne, et à qui on a administré de l'ocytocine au moment de l'opération, a été réalisé sur une période de trois mois. Les données comprennent les indications de césarienne, la posologie d'ocytocine, la désignation du clinicien prescripteur, le type d'anesthésie pratiqué pour la césarienne et l'estimation de la perte de sang.

**Résultats:**

Un total de 139 cas a été enregistré. La dose la plus courante était une perfusion de 20 UI (31.7%). La posologie potentiellement dangereuse de 10 UI d'ocytocine par injection intraveineuse a été utilisée dans 12.9% des césariennes. Des doses supplémentaires ont été utilisées chez 57 patientes (41%). Les trois premières indications pour la césarienne étaient la souffrance fœtale (36 patients, 24.5%), la dystocie (32 patients, 21.8%) et une précédente césarienne (25 patients, 17%). La perte de sang estimée était comprise entre 50 mL et 2000 mL.

**Conclusion:**

L'utilisation d'ocytocine au cours d'une césarienne dans le contexte local ne suit pas la pratique recommandée. Les conséquences sont potentiellement dommageables. Une éducation et des conseils fournis par des directives nationales avérées pourraient contribuer à atténuer le problème.

## Introduction

The United Nations’ Millennium Development Goals (MDG) initiative set specific targets to improve health and development in developing countries. The fifth MDG targets the reduction of maternal mortality by 75% by the year 2015.^1^ The leading causes of maternal deaths in Africa are haemorrhage (33.9%), infection (9.7%) and hypertensive disorders (9.1%).^2^ In South Africa, obstetric haemorrhage is the third commonest cause of maternal death.^3^ Reports from Botswana highlight that in 2009 a total of 86 maternal deaths were recorded in the country. The leading causes were obstetric haemorrhage (15.1%), unspecified HIV-related disease (12.8%) and genital tract or pelvic infections (8.1%).^4^

Botswana has a relatively high maternal mortality rate for a middle income country (330 per 100 000) and Botswana is not on a trajectory to meet the fifth MDG (38 per 100 000) despite 98% of deliveries occurring in health facilities across the country.^1, 5^ Factors that contribute to maternal morbidity and mortality need to be identified and managed in clinical practice to achieve the MDG.

Although oxytocin is widely used in obstetrics, it has been linked with maternal morbidity and mortality. The complications associated with oxytocin include fetal hypoxia, hyperstimulation of the uterus and uterine rupture.^6^ When given as an intravenous bolus, oxytocin causes transient hypotension, reflex tachycardia and an increase in cardiac output in a dose-related manner.^6, 7, 8, 9, 10, 11^

Apart from being a preventive measure, the use of oxytocin in the perioperative obstetric patient may paradoxically contribute to maternal morbidity and mortality.^12^ The recognition of the potential harm of oxytocin during childbirth has resulted in debate about the appropriate dosing of the drug. Recent research has identified the optimal intravenous dose that balances the risk of side effects with the benefit of preventing post-CS haemorrhage as 3–5 IU after delivery of the baby, as a single prophylactic dose for all cases.^13, 14, 15, 16^

The Royal College of Obstetricians and Gynaecologists and the National Institute for Health and Clinical Excellence recommend 5 IU intravenous bolus to maintain uterine contraction during caesarean delivery, consistent with the licensed dose for the drug.^17, 18, 19^ However, studies continue to provide evidence for use of even lower doses of oxytocin than those endorsed by current guidelines.^12, 13, 14, 15, 19^

Furthermore, there appears to be no consensus regarding the optimal infusion rate of oxytocin needed to prevent haemorrhage at CS.^8, 20, 21, 22^ The World Health Organisation (WHO) recommends infusing 20 IU in one litre of crystalloid giving 60 drops per minute over two hours as prophylaxis against bleeding caused by uterine atony during CS.^23^

A survey of 240 obstetric anaesthetists in the UK revealed that 87% of the 179 respondents gave 10 IU oxytocin at CS and 50% of them gave this by rapid bolus.^24^ A survey conducted in other countries demonstrated that up to 14% of clinicians used a 10 IU bolus oxytocin.^25^ The Confidential Enquiry into Maternal Deaths in the UK identified the use of 10 IU intravenously as a cause of death during the resuscitation of a hypovolaemic patient during CS in the period 1997–1999.^12, 26^ In South Africa, two maternal deaths have recently been linked to the use of high doses of oxytocin.^26^

### Key focus

The rates of CS are increasing internationally and are accompanied by a relative increase in the use of uterotonic drugs like oxytocin.^27, 28, 29, 30^ Although oxytocin is used routinely, there appears to be variation in its use beyond the recommended approach.^24, 25, 26, 31^ This variation is likely to pose a risk. It may contribute to maternal deaths and therefore remains a cause for concern.

The current standard of practice with regard to the use of oxytocin during CS at Princess Marina Hospital (PMH) has never been assessed. There are no protocols in place to guide prescribers. The aim of this study was to gauge the current standard of practice in the local setting against best practice and establish a baseline for recommending good clinical practice.

#### Objectives

Research on the use of oxytocin continues to reveal that historically high doses of more than 5 IU are not necessary for maintaining uterine contraction post-CS as lower doses are as effective. Lower doses offer the advantage of posing less side effects. The main objective of this study is to assess what the standard of practice is with regard to the use of oxytocin during CS in the local setting where medical officers, nurse and specialist anaesthetists, obstetricians and gynaecologists are involved in the use of the drug. This will be established by documenting the doses of oxytocin used during CS at PMH and the routes of administering these. Furthermore, the study seeks to establish and document the average estimated blood loss (EBL) during CS where oxytocin was prescribed at PMH and report the relationship of these two variables. The indications for CS where oxytocin was prescribed will be assessed. Additionally, the types of anaesthesia used during CS where oxytocin was also used is assessed. The standard of practice of oxytocin used around the time of CS is compared with international best practice guidelines as outlined above.

#### Contribution to field

The dangers of high oxytocin doses were highlighted following a maternal death in the UK in which oxytocin was a possible significant contributory. There has been pressure ever since to reduce the oxytocin dose and provide evidence of efficacy of a lower dose. There is a body of literature on oxytocin guidelines from the WHO and professional bodies, but there is little available on implementation and compliance with the guidelines. This paper attempts to fill this gap.

## Ethical consideration

Ethical approval was obtained from the Health Research Ethics Committee at the University of Stellenbosch and the Research Ethics Committees of the Botswana Ministry of Health and the PMH. A waiver of patient consent was obtained since the study only required examination of routinely maintained medical records.

## Method

### Study design

The study design is a clinical audit of current practice involving a case record review of routinely maintained medical records.

### Context of the study

PMH is one of three major public referral hospitals in Botswana. It is located in the capital city of Gaborone and provides essential obstetric service for a population of over 191 000 residents. Additionally, PMH receives referrals from primary and secondary health facilities in the southern part of Botswana, a catchment population of about one million people. In Botswana, the various clinicians directly involved with the use of oxytocin during CS include medical officers, anaesthetic nurses, obstetricians and anaesthetists. A dedicated obstetrics and gynaecology theatre is operational on a 24-hour basis catering for emergency and elective operations. Medical officers (non-specialists) in the department of obstetrics and gynaecology work on a rotational basis to perform CS at the hospital. Anaesthesia is similarly offered by the anaesthetic department's medical officers, anaesthetic nurses and specialists. On average, 128 CS are performed out of a total of 540 monthly deliveries at PMH.

All post-CS mothers are admitted to one ward with an average length of stay of about three days. Case records, including anaesthetic records, are kept in the ward until the patient is discharged.

### Selection and sampling of patients

The study was conducted between 01 March and 31 May 2011. Data were transcribed from case records of all women admitted to the post-natal ward following a CS done at PMH, where oxytocin was used during and after the operation.

### Data collection

We reviewed case notes of all post-CS patients admitted to the ward. These were identified from the admission register kept in the ward and cross-checked with the theatre record. The medication chart, operative notes and anaesthetic record were all reviewed. Only case notes where oxytocin had been prescribed were included.

A structured data collection form was developed to gather information on age, oxytocin dose, route of administration, whether further doses of uterotonics were used, indication for CS, whether the CS was elective or an emergency, type of anaesthesia, estimated blood loss (EBL) and prescriber's designation. The information was transcribed into the data collection form and later into an electronic spreadsheet (MS Excel).

Where there was more than one indication for CS recorded in the case notes, all were noted as separate indications in the results. Dystocia was taken to include cephalopelvic disproportion, delayed first and second stage of labour and failed vacuum extraction.

### Data analysis

Descriptive statistics such as frequencies, means and percentages were used for the analysis of the pooled data. Estimated blood loss and oxytocin dose were compared using regression and Spearman's correlation analysis.

## Results

A total of 139 mothers out of the 145 mothers prescribed oxytocin during CS were included in the study with a mean maternal age of 28.9 years (a range of 16–43 years). Six mothers were excluded due to insufficient documentation. Out of these operations, 24 (17.3%) were elective and 115 (82.7%) were emergency. General anaesthesia was the most popularly used, accounting for 64%, whilst spinal anaesthesia accounted for the other 36% (Table 1). For emergency CS, general anaesthesia was used in 81 per 115 (70.4%) of mothers compared to 8 per 24 (33.3%) of elective CS. Use of epidural anaesthesia was not reported.

**TABLE 1:** Type of anaesthesia in emergency and elective Caesarean section.

Anaesthesia	Elective (*N* = 24)		Emergency (*N* = 115)		Total (*N* =139)	
		
	*n*	%	*n*	%	*n*	%
Spinal	16	11.5	34	24.5	50	36.0
General	8	5.6	81	58.3	89	64.0

*N*, Given as total number; *n*, Given as number.

The top three indications for CS were fetal distress (36 patients, 24.5%), dystocia (32 patients, 21.8%) and a previous CS (25 patients, 17.0%) (Table 2). One CS was performed for poor maternal effort - a reason not usually recognized as a standard indication for CS. Where multiple indications were recorded, all were noted as separate indications and the percentage shown is out of all 147 indications. No maternal deaths were recorded during the study.

**TABLE 2:** Indications for Caesarean section (*N* = 147).

Indication	*n*	%
Fetal distress	36	24.5
Dystocia	32	21.8
Previous CS and/or Myectomy	25	17
Antepartum haemorrhage	13	8.8
Malposition (breech, transverse lie)	12	8.2
Failed induction of labour and/or Postdates	10	6.8
Eclampsia and/or Severe pre-eclampsia	6	4.1
Other (multiple fibroids, twins and/or poor maternal effort)	4	2.7
Cord prolapse	3	2
Bad obstetric history	2	1.4
Medical condition	2	1.4
Ruptured uterus	1	0.7
Intrauterine growth restriction	1	0.7

*N*, Given as total number; *n*, Given as number.

The indications for CS differed according to whether the CS was an emergency or elective operation (Tables 3 & Table 4). Percentages are calculated from the total number of indications recorded.

**TABLE 3:** Indications for emergency Caesarean section (*N* = 125).

Indication	*f*	%
Fetal distress	36	28.8
Dystocia	29	23.2
Previous Caesarean section in labour	14	11.2
Antepartum haemorrhage	13	10.4
Malposition	12	9.6
Eclampsia	6	4.8
Cord prolapse	3	2.4
Postdates	3	2.4
Failed Induction of labour	2	1.6
Medical condition	2	1.6
Ruptured uterus	1	0.8
Intrauterine growth restriction	1	0.8
Bad obstetric history	1	0.8
Twins	1	0.8
Poor maternal effort	1	0.8

**Total**	**125**	**100**

*N*, Given as total number; *n*, Given as number; *f*, Frequency.

**TABLE 4:** Indications for elective Caesarean section (*N* = 24).

Indication	*n*	%
Previous Caesarean section	12	50
Dystocia	6	25
Postdates	5	20.8
Bad obstetric history	1	4.2

**Total**	**24**	**100**

*N*, Given as total number; *n*, Given as number.

**FIGURE 1:**
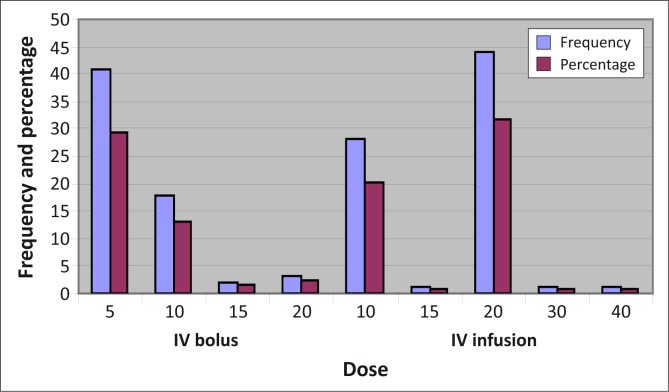
Different doses and routes of administration of oxytocin.

Different doses and routes of administration for oxytocin were observed (Table 5 & Figure 1). The commonest dose used was 20 IU intravenous infusion (31.7%).

**TABLE 5:** Different dose regimens of oxytocin used (*N* = 139).

Dose (IU)	Route	*n*	%
5	IV bolus	41	29.5
10	IV bolus	18	12.9
15	IV bolus	2	1.4
20	IV bolus	3	2.2
10	IV infusion	28	20.1
15	IV infusion	1	0.7
20	IV infusion	44	31.7
30	IV infusion	1	0.7
40	IV infusion	1	0.7

*N*, Given as total number; *n*, Given as number.

Further doses of uterotonics were utilized in 57 of the mothers (41%) and this included different doses of oxytocin and syntometrine.

Estimated blood loss ranged from 50 mL – 2000 mL. The mean blood loss and the corresponding oxytocin doses prescribed (see Table 6).

**TABLE 6:** Dose of oxytocin used and the estimated blood loss.

Dose	Mean blood loss (mL)	Blood loss range (mL)
5 IU IV bolus	382	100 - 1500
10 IU IV bolus	346	0 - 900
10 IU IV infusion	320	100 - 900
15 IU IV bolus	350	350
15 IU IV infusion	100	100 - 200
20 IU IV bolus	750	300 - 1200
20 IU IV Infusion	493	100 - 2000
30 IU IV infusion	500	500 - 1200
40 IU IV infusion	500	500

Prescribing practices differed widely, with medical officers and anaesthetic nurses accounting for all instances where the 10 IU intravenous bolus was used (Table 7). Anaesthetic nurses accounted for the three times where an intravenous bolus of 20 IU bolus was used.

**TABLE 7:** Oxytocin prescription by different categories of prescribers.

Dose	Prescriber	*f*
5 IU IV bolus	Anaesthetic nurse	7
	Medical officer	37
	O & G Specialist	1
	Anaesthetist	1
10 IU IV bolus	Anaesthetic nurse	2
	Medical officer	16
	O & G Specialist	0
	Anaesthetist	0
10 IU Infusion	Anaesthetic nurse	0
	Medical officer	25
	O & G specialist	0
	Anaesthetist	0
20 IU IV bolus	Anaesthetic nurse	3
	Medical officer	0
	O & G specialist	0
	Anaesthetist	0
30 IU Infusion	Anaesthetic nurse	0
	Medical officer	0
	O & G specialist	1
	Anaesthetist	0
40 IU Infusion	Anaesthetic nurse	0
	Medical officer	1
	O & G specialist	0
	Anaesthetist	0

*f*, Frequency.

**FIGURE 2:**
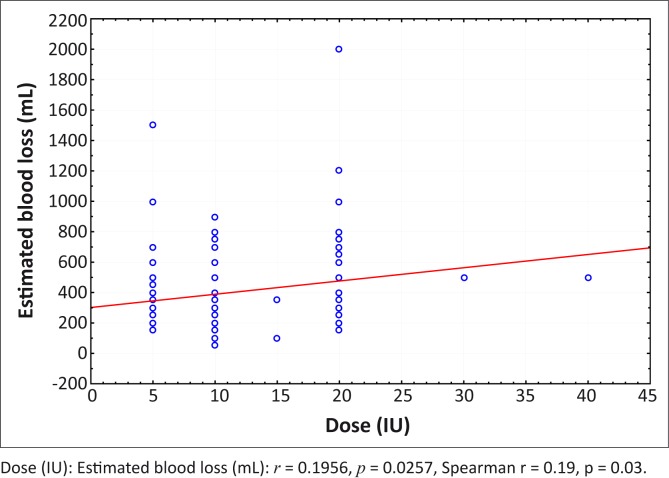
Dose (IU) of oxytocin vs. estimated blood loss (mL) for all regimens. Dose (IU): Estimated blood loss (mL): *r* = 0.1956, *p* = 0.0257, Spearman r = 0.19, p = 0.03.

There was a statistically significant relationship between the oxytocin dosage used and the estimated blood loss (*p* = 0.03), although the strength of the association was low (r = 0.19). The oxytocin dose increased with increasing estimated blood loss (Figure 2).

## Discussion

The present study revealed that only 30% of users adhered to the recommended practice of 5 IU intravenous oxytocin at CS. A total of 31.7% of participants in the study used an infusion of 20 IU in one litre of a crystalloid solution. As the infusion rates were not recorded, it was difficult to say if the WHO guideline was followed.

The low correlation of dosage and EBL suggests that choice of dosage is only weakly associated with the perception of blood loss and that variation in dose is most likely dependent on other factors. One would have expected a greater correlation between increasing dose of oxytocin and the higher EBL associated with post-partum haemorrhage in instances where an initial dose of oxytocin did not arrest the bleeding. However, this study did not differentiate between whether oxytocin was prescribed for prophylaxis against post-partum haemorrhage, or for its management once it had been diagnosed. Estimation of blood loss at CS is known to vary widely according to a clinician's perception and may have a large subjective element.^24^ Additionally, the skills and experience of the surgeon doing the CS is an important factor in the resulting blood loss from the surgery.

The number of oxytocin receptors expressed in the myometrium increases as pregnancy advances from 12 weeks gestation.^32^ Following continuous and prolonged exposure to oxytocin, there is significant loss in the capacity of the myometrial cells to respond as a result of oxytocin receptor desensitisation.^33, 34, 35^ It is in view of this physiological fact that there has been recommendations to consider other uterotonics where additional doses of oxytocin prove not to adequately control haemorrhage since these alternatives are not affected by the desensitation.^6, 26^

The high proportion of emergency to elective CS noted is likely due to the fact that high risk pregnancies are referred to PMH as emergencies.

Fetal distress (FD) was the commonest indication overall for CS and emergency CS where oxytocin was used, contributing 25% and 28.2% respectively. Although this study looked at a subset of women who were prescribed oxytocin during CS and not all women who had CS, the percentages regarding FD appear high. International studies suggest that rates for FD as an indication for CS were as low as 6% –17% in Ghana and as high as 46% in the UK for repeated emergency CS.^36, 37^

The significant contribution of FD as an indication for CS in our study begs the question of whether this entity is accurately diagnosed, since the diagnosis is made on observation of fetal heart rate on cardiotocograph. Despite the high sensitivity of cardiotocograph in detecting FD, it has a high rate of false positives, as well as inter- and intra-observer variation.^38^

The rates of general anaesthesia used during the study appear higher than in other African countries such as Nigeria, which reported a rate of 47.6%.^39^ The high uptake of general anaesthesia as opposed to regional anaesthesia in this study potentially increases the risk of anaesthesia-related causes of maternal morbidity and mortality since general anaesthesia is a recognised risk for most direct anaesthetic deaths.^40^

## Limitaions and strengths of the study

The unique setting of PMH that provides local district hospital type services and regional referral hospital services with a high volume of patients, presents a peculiar advantage in conducting this study. A variety of users were represented in the study with the majority being non-specialists - a situation closer in reality to the staff compliment of primary and district hospitals in Botswana.

The study did not identify specific individuals (but rather groups) who prescribed specific doses and thus potentially harmful practice could not be linked to specific individuals. However, this is useful in not creating a climate of blame - a clear objective of clinical audit.

The study could not distinguish whether oxytocin was primarily given as prophylaxis or treatment nor what the infusion rate was, since this was not stated in the case records from which data was transcribed. This highlights the fact that patient notes were not being adequately recorded. The deficiency in data could have been addressed better in a prospective study.

### Practical implications and recommendations

The effort to improve the current clinical practice with regard to the use of oxytocin during CS in the local setting, could include developing a national protocol for prophylactic use of oxytocin at CS. Junior doctors, anaesthetic nurses and non-specialist doctors should be targeted for continuing medical education activities that include training on the appropriate use of oxytocin at CS. This should also be addressed in the initial training of these clinicians and that of family physicians as they rotate through the departments of Obstetrics and Anaesthetics at PMH and other centres.

A re-audit of clinical practice after implementation of the protocol and appropriate education should be done.

## Conclusions

The use of oxytocin during CS in the local setting does not generally follow recommended practice and current literature. This has potentially harmful consequences, such as increased maternal morbidity and mortality. Education and guidance by national practice guidelines and local protocols could help alleviate the problem. This audit could be used as a baseline for future assessment of clinical practice at PMH and as a stimulus for similar clinical audits in other centres.
